# Can Continuous Local Anesthetic Infusion After Median Sternotomy Reduce Opioid Use?

**DOI:** 10.7759/cureus.10711

**Published:** 2020-09-29

**Authors:** Arda Aybars Pala, Yusuf Salim Urcun, Ömer Faruk Çiçek, Serpil Şahin

**Affiliations:** 1 Cardiovascular Surgery, Adıyaman Training and Research Hospital, Adıyaman, TUR; 2 Cardiovascular Surgery, Selçuk University Faculty of Medicine, Konya, TUR; 3 Cardiovascular Surgery, Canakkale Onsekiz Mart University Faculty of Medicine, Canakkale, TUR

**Keywords:** median sternotomy, postoperative pain, opioid, local anesthesia

## Abstract

Introduction

Effective treatment of postoperative pain due to median sternotomy speeds up hemodynamic healing of patients. For this purpose, opioids with a wide range of side effects are widely used at high doses. The aim of this study is to investigate the effect of continuous local anesthetic (bupivacaine) infusion on opioid use on cardiac surgery patients undergoing median sternotomy.

Methods

A total of 215 patients undergoing isolated coronary artery bypass grafting surgery were included in the study; and 105 patients who underwent parasternal continuous local anesthetic infusion (0.5% bupivacaine at 4 mL/h, for 48h) were determined as local anesthesia group and other patients were as control group. The primary outcomes evaluated between the groups in the postoperative period were pain scores (VAS: Visual Analogic Score, PHHPS: Prince Henry Hospital Pain Score) and the number of opioids used. Secondary outcomes were mechanical ventilation time, intensive care unit and hospital stay duration, development of atrial fibrillation and atelectasis.

Results

Postoperative pain was found to be significantly lower in the local anesthesia group compared to the control group (VAS: 3 ± 1.9 vs 6.4 ± 1.8, p < 0.001; PHHPS: 0.9 ± 0.8 vs 1.62 ± 0.82, p < 0.001). As a result of this, opioid drug use was significantly lower in the local anesthesia group compared to the control group (0 (0 - 4) vs 1 (0 - 8), p < 0.001). Mechanical ventilation time, intensive care unit and hospital stay duration, and development of atelectasis were significantly lower in the local anesthesia group. In terms of the development of atrial fibrillation, no significant difference was found between the groups.

Conclusion

Parasternal continuous local anesthetic infusion reduces postoperative opioid use and speeds up hemodynamic healing by preventing possible side effects of opioids. It is a simple and effective method in the treatment of postoperative pain due to median sternotomy.

## Introduction

Pain after coronary artery bypass grafting (CABG) surgery is mostly due to median sternotomy [1]. Pain, which is felt more severely in the first 48 hours postoperatively, may negatively affect the morbidity and mortality. Sympathetic nervous system activity increases due to postoperative pain and leads to increases in heart rate and blood pressure [2]. Accordingly, myocardial ischemia, cardiac insufficiency, and arrhythmias may develop as a result of increased myocardial oxygen requirement. Depending on the rise in blood pressure, the amount of bleeding in the early postoperative period may also increase. Besides, postoperative pain may cause difficulty in coughing in patients and related atelectasis and pulmonary dysfunction [2,3]. With the mobilization of patients, pain intensity can increase and prolong the healing process [4]. Effective treatment of postoperative pain accelerates the hemodynamic healing of patients, reduces morbidity and mortality, and indirectly leads to a reduction in intensive care and hospital stay, and healthcare expenses.

The most preferred anesthetic agents in the treatment of postoperative pain are opioids. Although it is known that opioids provide hemodynamic stability by reducing the stress response due to pain, side effects also pose problems in the healing processes. It is known that it can prolong extubation time, especially by causing respiratory depression and drowsiness at high doses and also may lead to addiction. It should also be noted that opioids have a narrow therapeutic window with side effects such as nausea, vomiting, constipation, itching, and bladder dysfunction [5,6]. Therefore, although they are beneficial in postoperative pain management, opioids should be used at minimum doses, times, and with caution [7].

As a result of understanding the importance of postoperative pain management after cardiac surgery, studies have been conducted on different analgesic and anesthetic agents administered to patients with different methods. However, the postoperative pain management strategy is still a subject under discussion. Our aim in this study is to investigate whether opioid use was affected by the continuous local anesthetic infusion on the sternum in the postoperative period in patients undergoing CABG surgery.

## Materials and methods

This study was conducted at the Department of Cardiovascular Surgery, Canakkale Onsekiz Mart University Faculty of Medicine, Canakkale, Turkey and included 215 patients who underwent CABG surgery with median sternotomy between January 2019 and January 2020. Data were retrospectively analyzed. Ethical approval was granted by the local ethics committee. The study was carried out following the principles of Declaration of Helsinki.

Among the patients who underwent CABG surgery with median sternotomy, 105 patients who received parasternal continuous local anesthetic infusion was defined as the local anesthesia group, and 110 patients who did not receive infusion were considered as the control group. The demographic, preoperative, perioperative, and early postoperative (the period until discharge) data of the patients were accessed from the hospital registration system. Age, gender, body mass index (BMI), diabetes mellitus (DM), chronic obstructive pulmonary disease (COPD), hypertension, EuroScore, New York Heart Association (NYHA) functional capacity classification, and ejection fraction (EF) were included as preoperative data. Aortic cross-clamp time and cardiopulmonary bypass time were evaluated as perioperative variables. Among the postoperative variables, mechanic ventilation time, atrial fibrillation development, number of opioids used, intensive care and hospital stay duration, body temperature, atelectasis development, and the pain scale scores were evaluated in the study.

The exclusion criteria were previous median sternotomy history, off-pump surgery, additional surgical intervention requirement other than CABG, emergency surgery conditions, neurological, renal or hepatic disease history, known local anesthetic drug allergy, postoperative intra-aortic balloon pump (IABP) need, high dose inotropic support requirement due to postoperative hemodynamic instability, prolonged mechanical ventilation support (> 48 hours), and re-exploration surgery due to bleeding.

Patients were premedicated by diazepam 5 mg orally the night before the surgery and morphine sulfate 10 mg intramuscularly 30 minutes before the start of the operation. After the patient was taken to the operating room, electrodes, venous and radial artery cannulas were placed; anesthesia induction was performed with fentanyl 30-50 μg/kg. Succinylcholine 1 mg/kg was used as a muscle relaxant and then pancuronium 0.1 mg/kg. During anesthesia maintenance, fentanyl 3 μg/kg/min infusion was used, and isoflurane inhalation if needed. Intubated patients were ventilated with 100% O2. CABG surgeries were conducted by the same surgical team with the same surgical method via median sternotomy to all patients. Left internal mammary artery and saphenous vein grafts were used for myocardial revascularization in all patients.

In the local anesthetic group, a multi-perforated, soft and small-diameter catheter was placed between the anterior face of the sternum and the subcutaneous fascia after the sternum was wired at the end of the surgery. The other end of the catheter was pulled out of the skin by creating a tunnel next to the incision line and was connected to an infusion pump (ON-Q® PainBuster® Pain Relief System, B. Braun, Hessen, Germany). This infusion pump has a 400ml volume disposable elastomeric reservoir and provides a continuous infusion of the infusion solution with a constant rate of 4 mL/h. The reservoir of the infusion pump was filled with 200 mL of bupivacaine 0.5% and applied for the first 48 hours postoperatively at a 4mL/h rate. After 48 hours, the catheter was removed. The reason for choosing bupivacaine as a local anesthetic drug was that it is one of the longest-acting local anesthetic drugs.

Visual Analog Scale (VAS) and Prince Henry Hospital Pain Score (PHHPS) pain scales were used to determine the severity of postoperative pain. The patients were trained on pain scales in the preoperative period. The VAS is used to determine the level of pain on a horizontal line in the range of 0-10 cm, no pain is defined as 0 in the scale, and 10 defines unbearable pain [8]. The PHHPS scale scores in five categories on a horizontal line: 0 = no pain on cough, 1 = pain on cough but not on a deep breath, 2 = pain on a deep breath but not on rest, 3 = slight pain at rest, and 4 = severe pain at rest [9]. The PHHPS scale is more widely used after thorax surgery. Still, since it evaluates painful symptoms and pain severity during basic physical activities such as breathing and coughing, we applied it to patients in our study groups. Both pain scales were applied to the patients in our study groups at the 12th hour postoperatively, and the observations were recorded in the patient's documentation.

Statistical analysis

The statistical analysis was performed using the SPSS software package version 11.5 (SPSS, Inc., Chicago, USA). Quantitative variables were expressed as mean ± standard deviation and median (minimum-maximum), and qualitative variables were expressed as the number of patients (percentage). The qualitative variable, which had two categories in terms of quantitative variables, was examined by using the Student's t-test if normal distribution assumptions were provided; if variables were not normally distributed, the Mann-Whitney U test was used. When the relationship between the two qualitative variables was examined, Chi-square and Fisher-exact tests were used. The statistical significance level was taken as 0.05.

## Results

A total of 215 patients who underwent isolated CABG surgery were included in the study. There was no statistically significant difference between the local anesthesia group and the control group in terms of demographic data (age, gender, BMI). When preoperative data such as diabetes mellitus, chronic obstructive pulmonary disease, hypertension, EuroScore, NYHA functional capacity classification, ejection fraction were evaluated, no statistically significant differences were found between the groups. The perioperative data (aortic cross-clamp time and cardiopulmonary bypass [CPB] time) also showed no statistically significant differences. This was an indication that similar surgical procedures with similar duration were applied to similar patient groups (Table 1).

**Table 1:** Demographic, preoperative and perioperative data BMI: body mass index, NYHA: New York Heart Association, XC: cross-clamp, CPB: cardiopulmonary bypass

	Control group (n = 110)	Local anesthesia group (n = 105)	p-value
Age (years)	63.1 ± 8.7	59.8 ± 12	0.13
Gender Male Female	78 (70.9%) 32 (29.1%)	73 (69.5%) 32 (30.5%)	0.82
BMI (kg/m^2^)	27.3 ± 4.3	26.8 ± 4.1	0.39
Diabetes mellitus	51 (46.4%)	42 (40%)	0.34
Chronic Obstructive Pulmonary Disease	29 (26.4%)	24 (22.9%)	0.55
Hypertension	64 (58.2%)	52 (49.5%)	0.2
EuroScore [median (min-max)]	1.6 (0.6-14)	1.25 (0.5-14)	0.35
Functional capacity (NYHA)	1.83 ± 0.69	1.93 ± 0.72	0.27
Ejection Fraction (%)	49.1 ± 8	50.9 ± 7.9	0.1
XC time (minutes)	76.1 ± 20.5	77.6 ± 23.1	0.56
CPB time (minutes)	114.9 ± 30.2	111.2 ± 37.8	0.42

**Figure 1:**
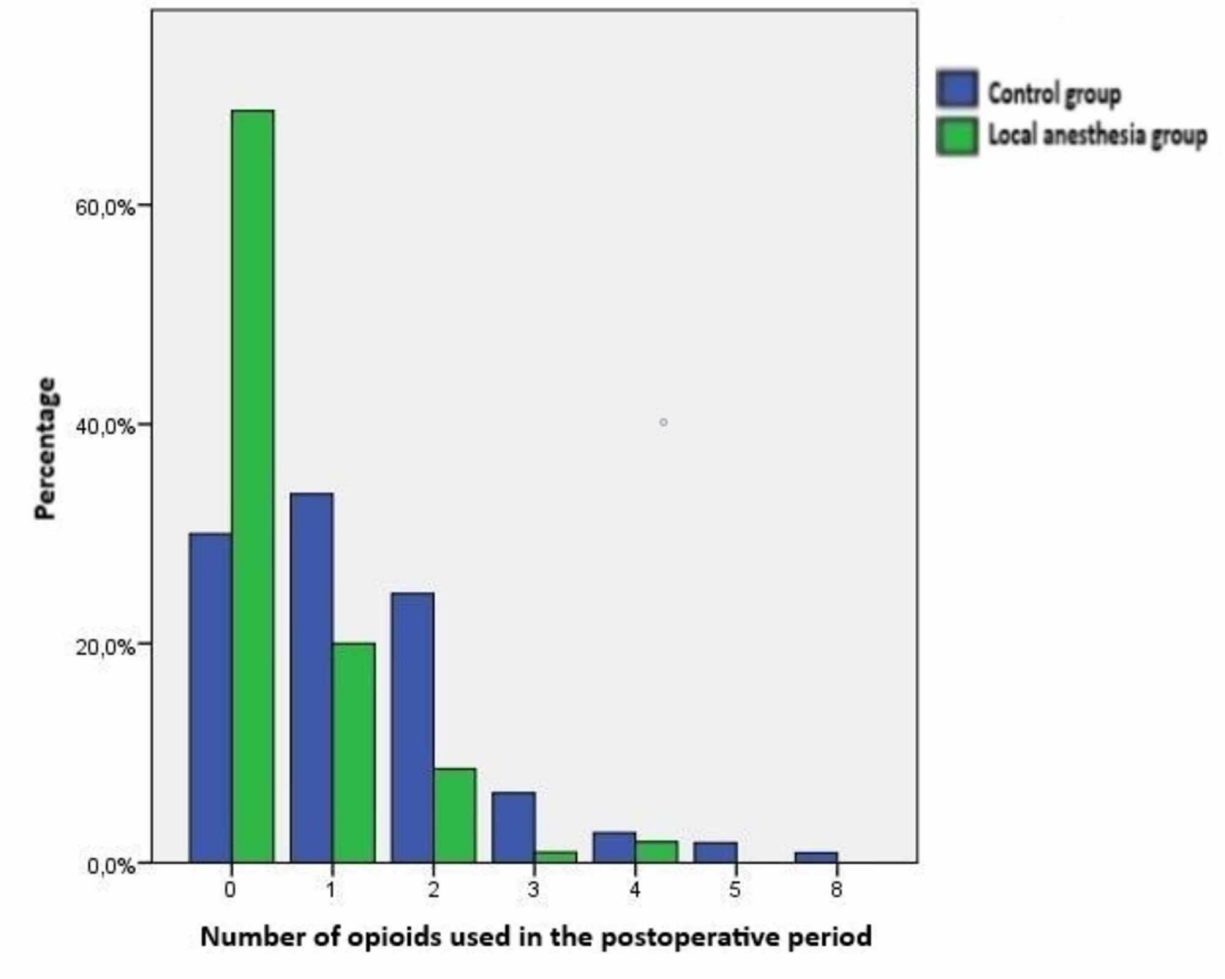
Distribution of number of opioids used in the postoperative period between groups

The main findings of our study emerged in the early postoperative period. First of all, it was seen that opioids were not used in the majority of the local anesthesia group (Figure 1).

When the number of opioids used in the early postoperative period was evaluated, it was found to be significantly lower in the local anesthesia group (p < 0.001). In addition, when the postoperative ventilation time, intensive care, and hospital stay of the two groups were compared, durations were found to be significantly lower in the local anesthesia group (p < 0.001). Atrial fibrillation development, which is the most common cardiac arrhythmia after CABG surgery, was found lower in the local anesthesia group; however, this difference did not create a statistical significance (10.5% vs 17.3%, p = 0.21). Atelectasis detected by posteroanterior (PA) chest X-ray examined at postoperative 24th hour was significantly lower in the local anesthesia group (18.1% vs 32.7%, p = 0.01). However, no statistically significant difference was found between the groups in body temperature measurements performed at the same hour (Table 2).

**Table 2:** Postoperative data ICU: intensive care unit, VAS: Visual Analogue Scale; PHHPS: Prince Henry Hospital Pain Score

	Control group (n = 110)	Local anesthesia group (n = 105)	p value
Postoperative ventilation time (hours)	12.5 ± 5.7	8.5 ± 2.9	< 0.001
Postoperative atrial fibrillation	19 (17.3%)	11 (10.5%)	0.21
Opioid number [median (min-max)]	1 (0-8)	0 (0-4)	< 0.001
Postoperative ICU stay (days)	2 ± 1.7	1.4 ± 0.8	< 0.001
Postoperative hospital stay (days)	7.8 ± 2.8	6.1 ± 1.7	< 0.001
Body Temperature (°C)	37.1 ± 0.45	37.1 ± 0.39	0.38
Atelectasis	36 (32.7%)	19 (18.1%)	0.01
VAS	6.4 ± 1.8	3 ± 1.9	< 0.001
PHHPS	1.62 ± 0.82	0.9 ± 0.8	< 0.001

**Figure 2:**
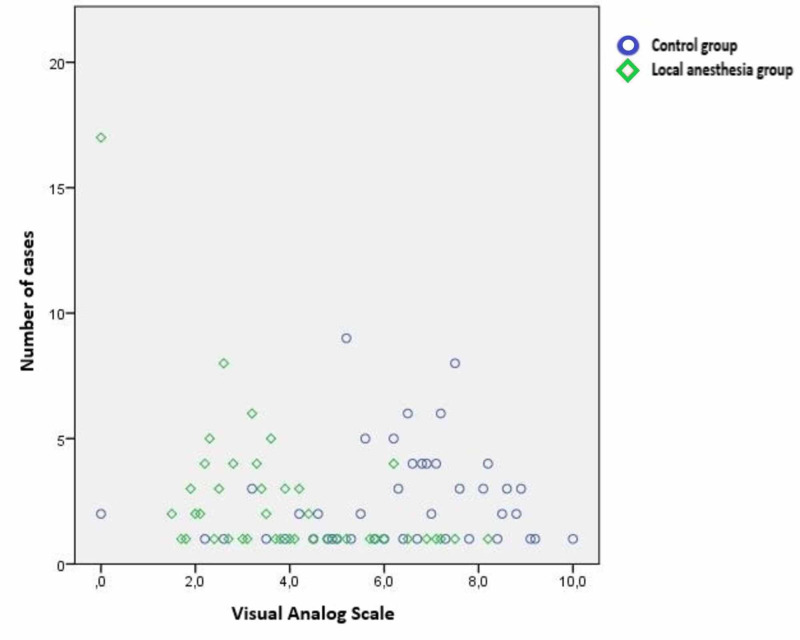
Distribution of Visual Analogue Scale scores between groups

**Figure 3:**
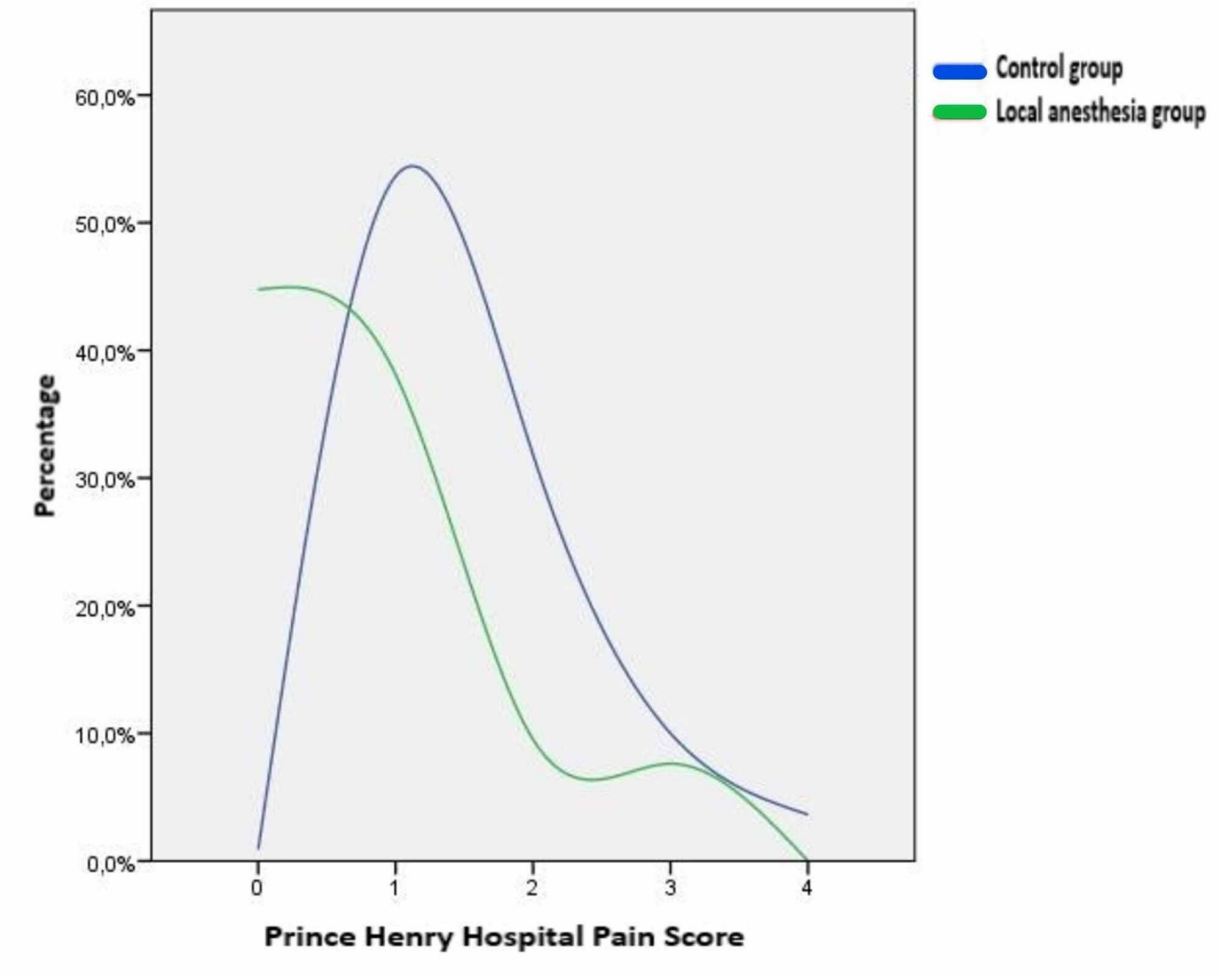
Comparison of Prince Henry Hospital Pain Score between groups

The pain levels of patients in the local anesthesia group were lower according to VAS and PHHPS scales that we applied at the 12th hour postoperatively to determine the pain intensity. (Figure 2, Figure 3). This difference between the pain intensities of the two groups was found to be statistically significant (p < 0.001) (Table 2).

## Discussion

Moderate or severe pain occurs in about 75% of patients after cardiac surgery, especially in the first 48 hours [10]. It is known that median sternotomy and associated skin incision, sternal retractions, and sternal wires play an essential role in the etiology of this pain. In the postoperative period, cardiac and pulmonary complications occur in patients due to pain and cause delayed hemodynamic recovery. For this reason, interest in minimally invasive approaches is growing in cardiac surgery today. However, the median sternotomy is still the most commonly used incision method in cardiac surgery.

Neural innervation of the sternum is provided by the anterior and posterior branches of the intercostal nerves. In cardiac surgery, as a result of direct trauma in the sternum, sensory stimulation of the primary afferent nerve terminals (peripheral sensitization) and changes in the central nervous system (central sensitization) occur in response to the released inflammatory mediators [11]. The complex structure of postoperative pain can be explained simply by this mechanism.

Postoperative pain severity can be reduced by preventing peripheral sensitization with local anesthetic drugs. For this purpose, there are studies on the infusion of these drugs into the wound area with different doses in different surgical procedures, and this method has been reported as an effective method for the treatment of postoperative pain [12-15]. In this study, we achieved better pain management with bupivacaine 0.5% (4 mL/h) infusion, which we continued to apply to the parasternal region in the early postoperative period, and reduced the use of opioids in patients who underwent median sternotomy.

Opioids are frequently used in intensive care units in the treatment of postoperative pain. The most commonly used opioid agent is morphine. Since the opioids have a narrow therapeutic window, undesirable side effects are often observed at high doses. For this reason, it has been observed that using different analgesic drugs and techniques in the treatment of postoperative pain and reducing the use of opioids accelerates the hemodynamic healing of patients. Wheatley et al. [16] reported that continuous local anesthetic (0.25% bupivacaine at 4 mL/h) infusion is an effective and safe method for postoperative pain management after thoracotomy. Koukis et al. [17] reported that continuous subcutaneous anesthetic (ropivacaine) infusion administered after median sternotomy reduced postoperative pain and opioid use. In our study, the rate of opioid use was found to be lower in the patient group in which we applied continuous local anesthetic infusion, and the difference between the two groups was found statistically significant.

The duration of the postoperative mechanic ventilator requirement was significantly shorter in patients in the local anesthetic group. This is thought to be due to higher opioid use rates in the control group and related opioid side effects such as respiratory depression and sedation. Nasr et al. [18] stated that the patients who received continuous parasternal bupivacaine infusion had better pulmonary functions, shorter extubation time, and reduced morphine use. In addition, patients were able to cough more easily after extubation with this analgesia method, and atelectasis rates were significantly lower in the PA chest radiographs evaluated at the postoperative 24th hour compared to the control group. In other words, the positive effect of continuous local anesthetic infusion on pulmonary functions continued even after extubation. This improvement in patients' pulmonary functions accelerated hemodynamic recovery due to more comfortable mobilization and significantly shortened intensive care unit (ICU) and hospital stay duration.

Atrial fibrillation is the most common cardiac arrhythmia, with an incidence of 20-40% in the early period (especially at 24-72 hours) after CABG. It is known that the most important cause of postoperative atrial fibrillation is tissue hypoxia [19]. In our study, the rate of postoperative atrial fibrillation development was found to be lower in the local anesthetic due to the better improvement results in pulmonary and hemodynamic functions, but this difference was not statistically significant.

Assessment of pain is a critical step to providing proper postoperative pain management. There are standardized and documented pain scales defined for this assessment. In the literature, studies report that continued infusion of different local anesthetics after median sternotomy reduces scores on pain scales [16,20]. In our study, we evaluated our patient groups with the widely used VAS and PHHPS scales that questions painful symptoms, especially in necessary physical activities. In the group which continued local anesthetic infusion was applied, pain scores were significantly lower in both pain scales.

There are many previously reported studies on continuous local anesthetic infusion after median sternotomy. However, there is no consensus on which anesthetic drug should be administered at which dose, in which period, at what infusion rate. In the future, more comprehensive and comparative studies are needed to determine optimal local anesthetic drug, drug dose, infusion rate, and duration for this analgesia method.

## Conclusions

In conclusion, continuous infusion of bupivacaine 0.5% (4 mL/h) after median sternotomy, with a simple catheter placed to the parasternal region, reduces postoperative pain and opioid use rate. With this easy-to-use and simple analgesia method, ICU and hospital stay durations are reduced based on accelerated hemodynamic recovery after cardiac surgery, and further, health expenses can be reduced due to these reasons.

## References

[1] Mueller XM, Tinguely F, Tevaearai HT, Revelly JP, Chiolero R, von Segesser LK (2000). Pain location, distribution, and intensity after cardiac surgery. Chest.

[2] Mazzeffi M, Khelemsky Y (2011). Poststernotomy pain: a clinical review. J Cardiothorac Vasc Anesth.

[3] Watt-Watson J, Stevens B, Katz J, Costello J, Reid GJ, David T (2004). Impact of preoperative education on pain outcomes after coronary artery bypass graft surgery. Pain.

[4] Bjornnes AK, Rustoen T, Lie I, Watt-Watson J, Leegaard M (2016). Pain characteristics and analgesic intake before and following cardiac surgery. Eur J Cardiovasc Nurs.

[5] Brook K, Bennett J, Desai SP (2017). The chemical history of morphine: an 8000-year journey, from resin to de-novo synthesis. J Anesth Hist.

[6] Mota FA, Marcolan JF, Pereira MH, Milanez AM, Dallan LA, Diccini S (2010). Comparison study of two different patient-controlled anesthesia regiments after cardiac surgery. Rev Bras Cir Cardiovasc.

[7] Roediger L, Larbuisson R, Lamy M (2006). New approaches and old controversies to postoperative pain control following cardiac surgery. Eur J Anaesthesiol.

[8] Breivik H, Borchgrevink PC, Allen SM (2008). Assessment of pain. Br J Anaesth.

[9] Zubrzycki M, Liebold A, Skrabal C (2018). Assessment and pathophysiology of pain in cardiac surgery: review. J Pain Res.

[10] Choiniere M, Watt-Watson J, Victor JC (2014). Prevalence of and risk factors for persistent postoperative nonanginal pain after cardiac surgery: a 2-year prospective multicentre study. CMAJ.

[11] Chapman CR, Tuckett RP, Song CW (2008). Pain and stress in a systems perspective: reciprocal neural, endocrine, and immune interactions. J Pain.

[12] Liang SS, Ying AJ, Affan ET (2019). Continuous local anaesthetic wound infusion for postoperative pain after midline laparotomy for colorectal resection in adults. Cochrane Database Syst Rev.

[13] Kuchalik J, Magnuson A, Lundin A, Gupta A (2017). Local infiltration analgesia or femoral nerve block for postoperative pain management in patients undergoing total hip arthroplasty: a randomized, double-blind study. Scand J Pain.

[14] Liu FF, Liu XM, Liu XY, Tang J, Jin L, Li WY, Zhang LD (2015). Postoperative continuous wound infusion of ropivacaine has comparable analgesic effects and fewer complications as compared to traditional patient-controlled analgesia with sufentanil in patients undergoing non-cardiac thoracotomy. Int J Clin Exp Med.

[15] Pontarelli EM, Matthews JA, Goodhue CJ, Stein JE (2013). On-Q ® pain pump versus epidural for postoperative analgesia in children. Pediatr Surg Int.

[16] Wheatley GH 3rd, Rosenbaum DH, Paul MC (2005). Improved pain management outcomes with continuous infusion of a local anesthetic after thoracotomy. J Thorac Cardiovasc Surg.

[17] Koukis I, Argiriou M, Dimakopoulou A, Panagiotakopoulos V, Theakos N, Charitos C (2008). Use of continuous subcutaneous anesthetic infusion in cardiac surgical patients after median sternotomy. J Cardiothorac Surg.

[18] Nasr DA, Abdelhamid HM, Mohsen M, Aly AH (2015). The analgesic efficacy of continuous presternal bupivacaine infusion through a single catheter after cardiac surgery. Ann Card Anaesth.

[19] Júnior MSB, Matkovski PD, Di Giovanni FJ, Fenili R, Varella EL, Dietrich A (2015). Incidence of postoperative atrial fibrillation in patients undergoing on-pump and off-pump coronary artery bypass grafting. Rev Bras Cir Cardiovasc.

[20] Kamel EZ, Abd-Elshafy SK, Sayed JA, Mostafa MM, Seddik MI (2018). Pain alleviation in patients undergoing cardiac surgery; presternal local anesthetic and magnesium infiltration versus conventional intravenous analgesia: a randomized double-blind study. Korean J Pain.

